# Patient and pharmacist perspectives on opioid misuse screening and brief interventions in community pharmacies

**DOI:** 10.1186/s13722-024-00460-y

**Published:** 2024-04-08

**Authors:** Deepika Rao, James H. Ford, Olayinka O. Shiyanbola

**Affiliations:** 1 School of Pharmacy, University of Wisconsin-Madison, 777 Highland Avenue, Madison, WI 53703 USA; 2 Center for Technology and Behavioral Health, Geisel School of Medicine, Dartmouth College, Hanover, NH 03755 USA

**Keywords:** Pharmacy, Opioid, Patient-centered, Screening, Brief intervention, Prevention, Implementation, Qualitative

## Abstract

**Background:**

Pharmacy-based screening and brief interventions (SBI) offer opportunities to identify opioid misuse and opioid safety risks and provide brief interventions that do not overly burden pharmacists. Currently, such interventions are being developed without patient input and in-depth contextual data and insufficient translation into practice. The purpose of this study is to qualitatively explore and compare patient and pharmacist perceptions and needs regarding a pharmacy-based opioid misuse SBI and to identify relevant SBI features and future implementation strategies.

**Methods:**

Using the Consolidated Framework for Implementation Research, we conducted semi-structured interviews with 8 patients and 11 pharmacists, to explore needs and barriers to participating in a pharmacy-based SBI. We recruited a purposive sample of English-speaking patients prescribed opioids for chronic or acute pain and pharmacists practicing in varied pharmacies (small independent, large-chain, specialty retail) settings. We used an inductive content analysis approach to analyze patient interview data. Then through a template analysis approach involving comparison of pharmacist and patient themes, we developed strategies for SBI implementation.

**Results:**

Most patient participants were white, older, described living in suburban areas, and were long-term opioid users. We identified template themes related to individual, interpersonal, intervention, and implementation factors and inferred applications for SBI design or potential SBI implementation strategies. We found that patients needed education on opioid safety and general opioid use, regardless of opioid use behaviors. Pharmacists described needing patient-centered training, protocols, and scripts to provide SBI. A short-self-reported screening and brief interventions including counseling, naloxone, and involving prescribers were discussed by both groups.

**Conclusions:**

Through this implementation-focused qualitative study, we identified patient needs such as opioid safety education delivered in a private and convenient format and pharmacist needs including training, workflow integration, protocols, and a time-efficient intervention for effective pharmacy-based SBI. Alternate formats of SBI using digital health technologies may be needed for effective implementation. Our findings can be used to develop patient-centered pharmacy-based SBI that can be implemented within actual pharmacy practice.

**Supplementary Information:**

The online version contains supplementary material available at 10.1186/s13722-024-00460-y.

## Introduction

Although opioid prescribing rates are decreasing, 16,706 overdose deaths involving a prescription opioid occurred in 2021 in the United States, a trend driven by combination with synthetic opioids such as fentanyl [1]. To take preventative actions to reduce overdose deaths and the risk of developing an opioid use disorder (OUD), healthcare professionals must recognize opioid misuse behaviors early. Efforts to address opioid misuse must not lead to inadequate pain management, especially among groups that receive disproportionately fewer opioid prescriptions, such as African American adults [2]. These issues can be addressed by leveraging community pharmacists who are highly accessible healthcare professionals, especially in rural areas with underinsured patients. Pharmacists have training in medication counseling, believe that screening for opioid misuse is important, and are interested in providing screening interventions [3, 4]. However, patients are not screened for opioid misuse behaviors when picking up their prescription opioids at the pharmacy. In the US, the role of the community pharmacist in OUD prevention and treatment has been mostly limited to dispensing medications for OUD and even then not at optimal levels [5]. There is a need to expand the role of the pharmacist in providing prevention interventions for OUD.

Nationally, calls to leverage community pharmacists as a resource in all types of OUD prevention, including screening and brief interventions (SBI), have increased [6]. Screening using prescription drug monitoring programs (PDMP) [7, 8] and brief interventions such as naloxone dispensing [9, 10] or opioid counseling [11] have been studied in pharmacy settings, but are rarely incorporated into one comprehensive SBI model [12]. Using a comprehensive SBI model to implement the interventions would increase their effectiveness and be more patient-centered. However, issues such as lack of clinical information and discomfort in talking to patients can act as intervention barriers [13].

We conducted a scoping review of pharmacy-based opioid misuse SBI and identified a few pilot-stage interventions and exploratory observational studies on this topic as well as two main research gaps [14]. While pharmacists were surveyed in development of these SBI, patient perspectives were not explored. Issues regarding private space, stigma, and method (in-person or digital) of the intervention as well as comfort with a pharmacist providing such interventions, all of which can impact SBI effectiveness, were not studied [14]. Patient-centered interventions that include individual patient preferences and values are holistic, respect patient’s autonomy, and empower them to make decisions about their own care [15]. Using a patient-centered approach to SBI development begins with exploring patient preferences and needs regarding participation. Our review also identified five qualitative studies that explored pharmacist perspectives regarding opioid misuse SBI but only one of the five studies had high credibility and trustworthiness [14]. There is a lack of in-depth, contextual information about pharmacist and patient perspectives of SBI, which is a significant limitation in development of effective interventions. Conducting qualitative exploration as the first step to designing the pharmacy-based opioid misuse SBI would help overcome this drawback.

To improve translation of SBI research into practice, it is useful to consider future implementation barriers at the design stage itself. The ‘designing for dissemination’ principles identify key actions in the process of designing interventions and the subsequent products [16]. These actions include engaging key stakeholders as early as possible, using implementation frameworks and dissemination constructs, documenting implementation barriers and outcomes [16]. Utilizing designing for dissemination and implementation principles at the development stage allows for more context-relevant interventions that addresses stakeholder needs and priorities.

The purpose of this study is to qualitatively explore and compare patient and pharmacist perceptions and needs regarding a pharmacy-based opioid misuse SBI and to identify relevant SBI features and future implementation strategies.

## Methods

### Consolidated framework for implementation research (CFIR)

The constructs under the CFIR domains that were appropriate for intervention design have been bolded (Additional file 1). The CFIR interview guide [17] was used to develop specific interview questions and the accompanying codebook template was used for initial deductive coding of interview data.

#### Study sample

Generally, 10–25 participants are considered sufficient for theory/model based qualitative studies using content analysis approaches [18, 19]. Our interviews had higher information power gained by sample specificity (purposive sampling [20] by targeting different pharmacy experiences and pain conditions rather than convenience sampling), using an applied conceptual framework (CFIR), the strong quality of dialogue (lengthy, in-depth interviews), and the exploratory nature of analysis (identifying patterns/themes rather than in-depth phenomenological description) [19]. Thus, interviews were conducted until data saturation was achieved, i.e. no new dimensions regarding the topic emerged [21] We conducted interviews with a purposive sample of adult, English speaking patients, living in a Midwestern state, who have been prescribed an opioid medication at least once in their lifetime for acute or chronic non-cancer pain. Patients diagnosed with an OUD, receiving opioids for cancer-related pain, or unable to participate in the interview (hospitalized, in hospice care, suffering from debilitating pain) were excluded from the sample. A purposive sample of English-speaking community pharmacists (those practicing in a non-clinical, community setting such as large national chain pharmacies, independent pharmacies, or specialty pharmacies) practicing in the same Midwestern state were included in the sample.

#### Data collection

For patients, recruitment initially occurred through regional pain clinics and primary care providers. To increase recruitment of individuals using in-person pharmacy services, pharmacists who completed study interviews were also asked to share study information with their patients. A study flyer describing interview procedures and other study information was sent to healthcare professionals to share with eligible patients. We briefed the healthcare professionals on the study purpose (i.e. exploring patient perspectives on SBI) and the larger goal of our research (i.e. designing a patient-centered opioid misuse SBI for pharmacy settings). We asked healthcare professionals to purposefully select individuals who may be good candidates for SBI. To recruit individuals with acute pain, we used the emergency department research coordinators. Patients were given the option to contact the study team themselves or allow their contact information to be shared with the study team. Pharmacists were recruited through emails sent to a practice-based research network and an informal list curated by the study team. Emails included study information and a screening and a contact information form.

Eligible and interested patients and pharmacists were contacted to schedule interviews conducted via telephone, web-conferencing software, or in-person. The lead researcher (DR) with training and prior experience in qualitative methods conducted all the interviews. Verbal informed consent was solicited prior to beginning the interview. The patient interviews were 30 min long and pharmacist interviews were 60-min long. Patients received $30 compensation and pharmacists received $50 compensation for completing interviews. All interviews were audio-recorded and transcribed, and transcriptions were used for further data analysis. The patient interviews focused on patient experiences in pharmacy and needs regarding their opioid medications in addition to the more SBI-specific questions. The pharmacist interviews were longer to accommodate additional questions focused on characteristics of their particular setting not directly related to patient care such as: organization goals and feedback, colleague networks and communication, and workplace culture. These data have been reported separately [22]. The interview guides were piloted in the first couple of interviews and probing questions were added as appropriate (ex. opioid experiences for patients, OUD prevention experiences for pharmacists). Patients were also prompted with examples of different types of interventions that pharmacists could potentially provide within the SBI model to generate richer discussions. The sample interview questions linked to the CFIR constructs for both pharmacists and patients are provided in Additional file 2. The Institutional Review Board at the author’s institution approved the study procedures after expedited review.

#### Data analysis

While a deductive analysis approach (based on CFIR) was planned initially, it was not suitable for the patient interview data as very little information could be coded using the CFIR constructs. Therefore, an inductive open coding approach was utilized for patient interviews. Two coders independently coded each interview transcript and discussed their coding in detail with DR as primary analyst and an undergraduate student as a secondary coder. Any conflicts in the coding were resolved at this stage. Finally, DR abstracted all categories into themes. Following content analysis of the patient interview data, a template approach was utilized to compare data from the patient and pharmacist interviews. The template was created based on the patient interviews first by listing the major themes resulting from the content analysis. Then pharmacist transcripts were analyzed using this template as the coding structure. Then, salient quotes from the two groups corresponding to the template themes were included in a matrix. This matrix was used to make comparisons and meta-inferences regarding pharmacists and patient perceptions of the SBI as well as report findings. Opposing views regarding the same themes across the two groups were also presented in the matrix. MAXQDA software was used for all qualitative analyses. DR created the template, conducted the analysis, and produced the matrix independently. Two researchers (OS, JF) who were not involved in the data collection process reviewed the final matrix to improve credibility and trustworthiness of the findings.

#### Rigor

Qualitative rigor was achieved by establishing credibility and confirmability through purposive sampling [20], achieving data saturation [21], using multiple coders for analysis (analyst triangulation), template analysis with patient and pharmacist data (triangulation of data sources), and peer debriefing [23]. The ‘Consolidated Criteria for Reporting Qualitative Studies (COREQ)’ checklist [24] has been completed for this study (Additional file 3).

## Results

### Sample characteristics

Eight semi-structured interviews were completed from May to October of 2021 virtually, over the phone, or face-to-face with patients taking opioid medications for non-cancer related acute (n = 2) or chronic (n = 6) pain. Patients used in-person pharmacy services, mail order, or drive through pharmacy services for their opioid medications. Most patient participants were white, older, and described living in suburban areas. Both men and women were recruited in the sample. Participants with chronic pain had used opioids consistently for 5–30 years, while participants with acute pain had used opioids after surgeries in the past 5 years. All participants were taking a combination of short -acting and long-acting opioids. While we did not ask participants to report opioid misuse behaviors directly, our recruitment method through healthcare professionals resulted in inclusion of individuals who had high opioid safety risks such as: requests for higher doses due to tolerance, development of hyperalgesia, family history of substance use disorders, possession of large quantities of unused opioids, and fills of prescriptions at different pharmacies. Eleven pharmacist interviews were completed from March to August of 2021 virtually. Pharmacists practiced in a variety of settings (large-chain, small independent, specialty) and roles (manager, owner, full-time, part-time pharmacist).

### Template analysis findings

The results of the template analysis are presented in Table 1. The template consists of 14 themes including individual factors such as experiences with opioids/care, knowledge, beliefs, needs, and self-efficacy interpersonal factors such as stigma and patient- pharmacist-provider relationships, intervention factors that describe beliefs and views on intervention components, and implementation factors such as implementation needs and challenges. The template themes, summaries of the themes from patient and pharmacists interviews, exemplar quotes, and our interpretations for applications to future SBI design and potential implementation strategies are included in Table 1.

**Table 1:** Template themes, representative quotes, explanation, and application for the SBI

Themes	Theme summaries	Exemplar patient quotes	Exemplar pharmacist quotes	Applications for SBI design
Experience with opioid medications/care	Patients had many years of experience with opioid medications but also had issues accessing opioids. While prescribers were using their clinical judgement to taper, patients did not trust them and did not believe they were dependent. Pharmacists were aware of these issues and tried addressing them by providing education, counseling about opioid safety and building trust	*“I haven’t had trouble until now because my primary doctor has been providing me prescriptions, but now trying to wean me off of it… I have been taking extended release and…I take short acting…she wants me to be off of it. But I had surgery and I'm recovering from that and…I need it more.”*—Pt 2 *“It sucks that they keep trying to decrease the medication I'm on. [Prescriber] just says something about the pain medication causes you to have more pain than you're really in. I don’t think it does.”*—Pt 4	*“There's a lot of people that take narcotics on a regular basis that don’t think there is a problem where maybe there is a problem. –*RPh 11 *“Whenever you talk to them about their controlled substances, they get defensive. But I think over time, once you explain our system and why it's important, you can build the rapport and trust with them.*”—RPh 4 *“If you're finding that you're needing more of it to basically treat the same type of issue, at that point we invite them to start talking to us or to start talking to their doctors…It's [opioids] there for a purpose, and we do want you to use it, we just want you to use it safely. So, that's how we spread our message to help combat and answer questions about possible opioid misuse*.” -RPh 8	SBI should not be perceived as another barrier to accessing opioids, rather as an opportunity to receive more education about opioid use and safety
Knowledge and education about opioid safety	Patients did not have much knowledge of opioid safety. They described being directed to ‘take as intended’ but were not counseled regarding opioid safety. Pharmacists agreed that patient counseling provides opportunity to discuss chronic opioid use, misuse and opioid safety	*“[All I was told about opioid safety is] that you should do what the bottle says, and not overuse it.”*—Pt 02 *“I had back surgery eight months ago, and I was prescribed…70 hydrocodone, and I still have 50 left…If I’m prescribed opiates [again], and I run out of those, can I use what I have left from the others?”*—Pt 7	*“We have these conversations with patients regarding refill too soon or usage of opioids and how often they should be using them at least every other week, if not more.”-*RPh5 *“We would use it as an educational opportunity to show patients how an opioid will actually work in the system for chronic use.”.”*—RPh 10	BI must include patient education on general opioid safety
Beliefs about opioid safety, OUD	Patients strongly believed that they were not at risk of opioid misuse or developing dependence/ OUD. They believed that opioid misuse was common, but only among people who used drugs recreationally. Pharmacists were aware of these limiting beliefs and wanted to provide education address them	*“I think that the opioid epidemic or whatever they want to call it, they make such a fuss about it. But I believe that it has a lot to do with the recreational drugs that people take, the alcohol intake. Where, myself, I don’t fit into that…My brain doesn’t work like an addict.”*—Pt 7 *“I don’t have the whatever in me that gets addicted to things, because some people do and some people don't. It's just like alcoholism.”* –Pt 1	*“Letting them know that overdoses are never planned. I've heard that so many times. Well, “Yeah, that's not going to happen to me, I'm not that kind of person”, there's, so many stigmas related to that. So, trying to break down some of those statements and just give education. I don’t think patients realize what risks are really out there all day.”—RPh 6*	BI must address common patient misperceptions and limiting beliefs related to OUD and naloxone to improve opioid safety behaviors
Opioid care needs	Patients described various needs for people using opioid medications including counseling regarding non-opioid medications or pain management alternatives, more education and information about recognizing tolerance and dependence, how to handle an accidental overdose, contra-indicated substances, consequences of intentional misuse including legal issues, and in general more patient-centered counseling about opioids. Pharmacists also believed that patients needed opioid education, which could be met through SBI	*“I'd be lying to you if I didn’t joke with people and say: Hey, I've got this prescription. Now, how much money can I make on the street? I would never do that. But other people might, and they need to know what the issues could be.”—*Pt 8 *“I don’t know if there’s such a thing as being more addicted to something when you’re already on [opioids], drinking alcohol and recreational drugs. But maybe a little more information on that.”—*Pt 7 *“If they're really, truly in pain and they need this prescription, they would be more than willing…to try to get a better understanding of what the pain medication is going to do.”—*Pt 3	*“For the short term [acute pain], their needs are probably best met with some education that there can be risks with these [opioids], take the minimum amount of medication that you need to help you, when you don’t need it anymore, destroy it, get it out of the house. For chronic pain patients, long-term, knowing that tolerance is very common, that doses tend to escalate, that there can be drug interactions to watch for, things can increase the risk of affecting your breathing.”—*RPh 3 *“A lot of people will bundle the possibility of dependency versus addiction, and that's really not the case.”*—RPh 8 *“We would use it [SBI] as an educational opportunity, to show patients how an opioid will actually work in the system for chronic use.”*—RPh 10	BI can be beneficial to patients regardless of misuse if information on opioids, long-term opioid use (including concepts such as dependence, tolerance, sedation, and hyperalgesia), and overdose prevention are included
Self-efficacy	Patients had confidence in their ability to take opioids safely because they have been taking it for many years. Hence, they believed the SBI would be more useful for new patients and not for experienced patients. Pharmacists had high self-efficacy to deliver SBI, especially if they had existing relationship with their patients. They also believed first-time prescriptions were the greatest opportunity to deliver SBI	*“I'm very confident because I've taken it for so long without having any issues.”*—Pt 1 *“I’ve taken them [opioids] for years and that I, without them, I can’t function. And I feel comfortable because my doctor explains anything that I have questions about.”*—Pt 6 *“I'm doing well, and I don’t need it [SBI] because I've already asked all the questions.”*—Pt 4	*“I have a pretty good relationship with my patients, so I would be able to talk with them, with empathy and understanding and that they would understand where I'm coming from, and that it's not accusing them of anything, but it's a matter of safety.”*—RPh 3 *“Whether or not they're getting this opioid for the very first time, because that presents the greatest opportunity for us to talk about the issue and everything with dependency or misuse.”*—RPh 8 *“Oh, incredibly confident. We’ve been doing it for years. That easy access—we’re the point person in healthcare, so people do have that conversation, for sure.”*—RPh 1	SBI may be ideally delivered at index prescription, but long-term relationships with patients can make pharmacists more comfortable in delivering SBI
Stigma	While only some pharmacists admitted to being biased towards patients picking up opioid prescriptions, most patients discussed feeling stigmatized by healthcare professionals, which is a barrier to SBI participation	*“It feels like every time you get an opioid medication, you're being looked at like you're an abuser, or like does this person really need it?”*—Pt 1 *“There was one time when I was at a local pharmacy… And he [pharmacist] treated me like I was a drug addict. And so I just quit going to him… he just, he looked at me, and he’s like, “Boy, this is a lot of medicine and for someone so young. Do you really need all of this?” And it was very discomforting.”*—Pt 6	*“I'm sorry but I have to bring some bias to some of this because I want to be aware of the entire situation and have that gut feelings saying, “Hey is this somebody who's maybe abusing? Are they telling me the whole story?”*—RPh 6 *“We have a lot of patients who want to fill their controlled substances up just a couple of days early…I think my coworkers are skeptical sometimes, of those situations… especially with opioids or other C2’s”*—RPh 2	Patient centered education, anti-bias training to address stigma against OUD may be necessary
Patient –pharmacist—prescriber relationships	Patients used informal sources such as the internet for questions about their medications. They also had conversations with prescribers about their medications but most never discussed it with their pharmacists and did not view pharmacists as providers of clinical services. While many pharmacists attempted to intervene when they suspected misuse, some were not comfortable with it and did not view it as part of their practice scope	*“…at some point, you would think the doctor could realize [recognize misuse]. Because I know my doctor takes steps…a lot of doctors are very good at telling that, seeing in a patient, whether they really need them [opioids] or they don’t. That’s what doctors get trained on. I truly think that it would be more up to the doctor in the 1st place, because he's the 1 who's going to prescribe it.”*—Pt 3 *“I research it [opioid medications] online, or I'll ask my general practitioner.”*—Pt 5	*“We have a few patients; they'll get five-day prescriptions for hydrocodone. And it'll be from a couple of different doctors sometimes it'll be one every six hours [or] it’ll be one every four [or] every eight, and it’s odd to me. So, I do try to delve into, like: “Hey,… what's still going on that you still need these three-day courses… couple times a month they'll get a couple of days of it. And I don’t really get anywhere because I am afraid of them jumping to conclusions.”*—RPh 2	SBI may need to be marketed as a clinical service. Strategies may include advertising SBI and clinical role of pharmacist using posters and brochures that prompt patients to ask questions
Beliefs about SBI	Both patients and pharmacists believed SBI could be helpful in providing patient education regarding opioid safety and misuse. While patients would like more information about their prescription, they did not want to be told what to do or the pharmacist to ‘interfere’. Some patients also stated that people may not be honest about misuse behaviors. Pharmacists would like training in improving their comfort with screening for and providing counseling on such a sensitive subject and making it into their routine practice or policy for opioid medications	*“I'm good with it [SBI]. I think my pharmacy is amazing. I prefer to know about the medications I'm taking. I mean, you're only helping yourself when they give you this information… my pharmacist does that [counseling]already. He’s very knowledgeable, he says if, if this [fentanyl]patch is working for you and you can go longer without your pills, then maybe we could cut down on the pills… he's already giving this information.”* –Pt 3 *“I think most people would think it [SBI] would be fine. But then some people would feel like it's interfering. So maybe for those patients, it would be helpful to tell them that this is just information only.”*—Pt 4 *“It all depends on the status of the person that's taking the opioids. If they're abusing them, I don’t feel they're going to be very open to any type of counseling, or they might not be honest, but I would hope that they would perceive those 3 or 4 questions as the pharmacist being concerned.”* -Pt 5	*“I'm pretty confident that we are at the very least get some momentum and set the groundwork for [SBI], what could evolve into this standard of practice.”*—RPh 10 *“I don’t want to be coming across like I'm accusing this person of being an abuser of medication. I don't seem to have a problem with it [counseling], because we do a lot of the homework and prep work ahead of time. As far as: “Hey, this is our policy about filling these prescriptions”… [SBI needs include] training more on making pharmacists feel comfortable that they can discuss that issue with patients. I think that's it.”*—RPh 8 *“I could really see this [SBI] being perceived as I’m now receiving better, personal touch, clinical care that I would never have expected.”*- RPh 10	SBI must be patient-centered and provide information without using accusatory or labeling language. Pharmacist training and introducing SBI as personalized clinical care into routine practice may be helpful
Screening component	Patients discussed answering screening questions on a tablet, form, or app for privacy or wanting a face-to-face conversation with pharmacists. Pharmacists wanted the screening to be in addition to services already offered. They also discussed needing technician help to initiate the screening through a form or tablet. Some suggested doing the screening over the phone to make it more efficient and save pharmacist time	*“I think an interactive tablet might be good. A form, obviously, you can certainly do that. But a form might, given all the germs and everything like that, form might actually be better than a tablet.”*—Pt 1 *“I think both [in-person and digital] would work. What would I prefer? Probably talking to the pharmacist.”*—Pt 4	*“The ideal screening would start with some of the things that were already just doing by default—checking the PDMP, calculating the morphine equivalence for everything, looking for diagnosis codes for what an opioid is being prescribed. We are assessing the risk of the other medications that they're on that would increase the risk such as concurrent benzos. That'd be part of our initial information gathering prior to any kind of screen.”*—RPh 10 *“You would have to involve the entire pharmacy. So, that technicians would be able to initiate the whole process, they'd recognize this is a controlled substance, and we have not talked with this patient before.”—*RPh 6	Online, phone, and in-person formats of screening were suggested but opinions on feaibiliy and patient preferences varied.. Standardized screening tools may be used if they are brief (< 5 min) and easy to answer
Brief Intervention—Naloxone	Patients and pharmacists discussed naloxone as a potential brief intervention. Many patients did not think they needed it because they believed it was only for people who intentionally misused opioids. There were knowledge gaps as well such as being able to administer naloxone to themselves. Pharmacists wanted a script where naloxone is antidote for a potential side-effect of the opioid rather than patient’s intentional misuse behavior	*“I think that the doctors are starting to, give counter measures so if people would accidentally OD they could help themselves at home with Narcan. And I think that’s good and that maybe people understand and know how to use that.”*—Pt 6 *“I'm afraid that if people could get narcan to carry with them…it’s giving them a reason to take more, because they could use that [naloxone] and it'll bring them back. I think that could go either way.”*—Pt 3	*“making it maybe a little bit easier to dispense [Narcan] sometimes. So, having a script to explain what it is, possibly having a script to say why we're dispensing it.”*—RPh 5 *“Everyone seems to understand the concept of an Epi-pen, we usually explain the Narcan is like an Epi-pen for an overdose.”*—RPh 1	Naloxone counslign and dispensing can be potential BI but a non-stigmatizing script for pharmacists may be needed
Brief Intervention—Counseling	Patients were enthusiastic about BI if it improved their knowledge of opioid medication safety. They suggested both face-to-face conversations or online digital app-based education options for BI. Either way patients stressed the need for autonomy in the design of SBI. Pharmacists also suggested that counseling could be used as BI to improve patient knowledge on opioid use and safety. Handouts could be used to help with counseling and reduce the time needed	*“Just ensuring people that they're [pharmacists] there for information, pretty much information only and that they're not telling them exactly that they have to change anything that they're doing, just being there for more knowledge and then leaving it up to them if they're going to change. Because when people have more knowledge, they're more willing to change things about what they're doing than just to be told, well, you should be doing it this way.”*—Pt 04 *“I think before you can accept or decline the prescription that you have to read through the information, which maybe people will, maybe people won’t. But at least you get, have a little box that says I acknowledge something.”*—Pt 7	*“My philosophy as far as counseling patients is not as strict as some other pharmacists, but I really truly believe in a much more collaborative type of interaction.”* -RPh 11 *“Educating people on opioids and the potential for misuse and Narcan, those are very attainable goals with a screening tool and an intervention.”—*RPh 02 *“To provide every patient who is getting an opioid prescription, locations of where medication can be discarded once it's done. More than just saying something to them, now we have a handout that we give to our patients.*—RPh 10	Counseling as potential BI offers ample opportunity for patient education but the counseling offered must be patient –centered. Digital formats of SBI were suggested to provide this education
Brief Intervention—Contacting prescribers	Patients were comfortable with their pharmacist contacting prescribers regarding opioid medications. However, they wanted to be involved in that process and be aware of the conversation. Pharmacists also suggested that contacting prescribers regarding inappropriate opioid prescriptions must be done but with the patient’s approval	*“I don’t know if currently there is a system or a program or procedures in place where pharmacists can have actual conversations with doctors. I don't know if that's a regular thing, if they regularly do. But I think that's not a bad thing if somebody feels that this stuff is just too strong, I don't need this much. Or it's not doing enough, because there might be a different medication or a higher dose or something along those lines that might be better for that patient. I think that that kind of conversation should be happening.”*—Pt 1	*“Let the patient know why you have reservations and then let them know what you're doing—contacting the doctor. Maybe the doctor's not aware. I've had a couple instances mostly with our ER doctors where they will prescribe pretty strong pain reliever, it's really potent medications in people who probably may or may not need them or the doses. So, they may or may not be aware of the history or other drugs that the person is taking. So if you see something that's not appropriate you'd want to contact the provider and discuss whether you should proceed, or whether they should try something different.”*—RPh 7	Pharmacists can contact prescribers as part of BI as long as patients are involved in the process
Implementation Needs	Patients wanted education in a format that offered autonomy and privacy. They also wanted the prescriber to be involved. Pharmacists discussed needing a protocol (instead of relying on judgement) and training to provide the SBI. They also discussed prescriber education and involving prescribers as stakeholders in SBI implementation to get their buy-in	*“Offering the benefit of what the program does, and then ask them, would you like to participate?…that's the best way to do it.”*—Pt 1 *“Face to face is the best. I think it forces the patients to communicate, to think about it because you have someone in your immediate presence as opposed to filling out a form, or even on a phone call where you can let your mind wander.”*—Pt 8 *“The world is going to cell phones and computers, so just somehow get information out on there…Maybe a pharmacist could send a text out and say, your refill is due in four days, and at that time, have a little skit that tells you about the opioid before you accept it.”*—Pt 7 *“I think the doctor and the pharmacists both need to have a discussion with this person.”*—Pt 3	*“My biggest problem right now—is making the professional judgment of when I should do this or when I should not.”*—RPh 8 *“There would have to be an education piece for pharmacists there. I don’t think I'm alone in saying that that I would be out of my comfort level.”*—RPh 6 *“Having communication with providers…there's the providers that don't care at all about it and are like: “Well, my patient’s in pain. I need them on as many pain meds as they can. Who are you to question me?” And then you have the providers that are like: “Well, I'm being judged now, so my patient's going to get nothing, and they're left with no meds.” And then those patients end up using drugs on the street or heroin or things like that.”*—RPh 9	SBI may need multiple formats (face-to-face /online) to offer patients an individualized service. Prescribers must be involved in implementation as stakeholders. Educational material and training for pharmacists must also include a protocol for providing SBI
Implementation challenges—Time, Roles & Stigma/ Privacy	Both patients and pharmacists identified time required as the primary challenge for SBI. Some patients did not perceive that pharmacists’ role to provide SBI and pharmacists had similar concerns regarding their ability or scope of practice. Patients did not want have conversations without privacy and pharmacists did not want to be perceived as accusatory. Pharmacists suggested building rapport focused on patient autonomy to avoid perceptions of stigma/interference	*“There's a certain amount of embarrassment that “I don't know what's going on,” or “I didn't listen to the prescribing doctor.” I think that's something that has to be taken into consideration. Time is an issue. Where do you find the time?”*—Pt 8 *“I like to have a relationship between my doctor and myself rather than the pharmacist. And when you go to the pharmacy,… I don’t know if people would be comfortable answering questions when there’s five people standing there listening to you.”*—Pt 7	*“I think some of the challenges we're going to face one is going to be time pressure. Two is going to be feeling like, perhaps you're not educated to really ask these questions and make the interventions. And I think three is going to be a certain fear that you're going to be perceived as somebody who is now the accuser of the patient.”*—RPh 10 *“One thing that helps is having a good rapport with the patient. If you come off the first interaction with that patient basically trying to be their parent on how they should take their medication, that rapport might not be very good.”*—RPh 4	Using digital formats may provide more privacy and save time. Appropriate patient centered SBI training for pharmacists and marketing it as a clinical service may help improve pharmacist roles and reduce stigma

*OUD* Opioid Use Disorder, *SBI* Screening and Brief Interventions, *BI* Brief Interventions

### Summary of results

Overall, we identified the following key findings related to individual, interpersonal, intervention, and implementation factors.

#### Individual factors

1. Experience with Opioids/Care: While providers used clinical judgement to taper opioids, patients did not trust them and perceived it as an access barrier to medications. Pharmacists were aware of these issues and tried providing education and counseling to patients. These findings indicate that patients may perceive SBI as another barrier to accessing medications rather than an opportunity to receive education about opioid use and safety.
2. Knowledge about Opioid safety: Patients had large knowledge gaps regarding opioid use (especially long-term use), opioid dependence, and opioid safety and the only directions given to them were to ‘take as prescribed’. Pharmacists were aware of these gaps and believed patient counseling would help. Therefore, patient education on chronic opioid use and opioid safety are important for the design of brief interventions (BI).
3. Beliefs about Opioid Safety and OUD: Patients believed that they were not at risk of opioid misuse, overdose, or developing OUD because it occurred among people who used opioids recreationally only. Pharmacists described these beliefs as barriers to opioid safety and naloxone dispensing. Addressing such common misperceptions and beliefs should be part of SBI design.
4. Opioid Care Needs: Patients discussed needs including recognizing tolerance, dependence, consequences of intentional and unintended misuse, non-opioid alternatives, managing an accidental overdose, and contra-indicated substances. Pharmacists suggested additional topics including pain management expectations and risk of addiction or accidental overdose, especially in patients who are older, are co-prescribed other medications, or have co-morbid conditions. Pharmacists also believed BI could help deliver this much-needed education. This indicates that BI could be beneficial to patients regardless of opioid misuse behaviors if education on long-term opioid use is included.
5. Self-efficacy: Patients’ confidence in taking opioids safely due to many years of experience made them reluctant to participate in SBI, indicating that SBI may be ideally delivered at index prescription. While pharmacists agreed with this, they had higher self-efficacy in providing SBI for established patients than new patients.

#### Interpersonal factors

1. Stigma: While only some pharmacists described being biased towards patients using opioids, most patients perceived stigma from healthcare professionals including pharmacists. This is huge barrier to potential SBI participation. Patient centered education and anti-bias training to address stigma against OUD may be necessary for pharmacists.
2. Patient-Pharmacist-Prescriber Relationships: Patients used informal sources such as the internet for medication questions or talked to prescribers rather than pharmacists who were not viewed as clinical healthcare providers. Some pharmacists had reservations about counselling patients. Pharmacists in our sample indicated they needed training. To overcome these role perceptions, marketing SBI as a clinical service is a potential implementation strategy that will need to be tested in future research.

#### Intervention factors

1. Beliefs about SBI: Despite the interpersonal challenges discussed above, patients were interested in pharmacy based SBI as long as it was focused on patient autonomy. While all patients found a short screening acceptable, some patients stated that individuals may not self-report opioid misuse. Their motivation to participate was primarily to obtain education about opioid use and safety. Pharmacists believed SBI would be helpful but were wary of stigmatizing the patients. Pharmacists described needing training and a protocol to provide SBI such that the interventions are integrated into routine care. They also suggested introducing SBI as personalized clinical care.
2. Screening Component: Both groups recommended short (< 5 min), standardized, self-reported screening and discussed potential screening formats such as online, in-person, or telephonic methods. Pharmacists also suggested using pharmacy technicians to help conduct the screening. However, feasibility perceptions among pharmacists and patient preferences for these methods varied.
3. Brief Intervention Components: Naloxone dispensing, patient counseling, and contacting prescribers (with non-stigmatizing scripts, handouts, and protocols) were discussed as potential BI.

#### Implementation factors

1. Implementation Needs: Patients wanted SBI implemented in a manner that offered privacy and autonomy. Multiple formats of SBI may be needed to offer patients the individualized service they are seeking. Pharmacists needed training and protocols that fit within workflow. If contacting prescribers were part of SBI, pharmacists suggested engaging prescribers as stakeholders.
2. Implementation Challenges: Three potential implementation challenges that were discussed included time, stigma, and pharmacist roles. Both patients and pharmacists were interested in an intervention no longer than 15 min. Alternate formats and using technicians may help reduce time burden. Offering SBI in a private space where available, integrating SBI into telehealth services, or using digital health technologies could potentially provide privacy and reduce perceived stigma. Marketing SBI as a clinical service provided by pharmacists and involving prescribers as stakeholders may help address pharmacist role challenges.

## Discussion

Our study is an initial exploration of pharmacist and patient needs regarding opioid misuse SBI for pharmacy settings. A short-self-reported screening and brief interventions including counseling, naloxone, and involving prescribers were discussed by both groups. We found that patients needed education on opioid safety and general opioid use in a private and convenient format, regardless of opioid use behaviors. Pharmacists described needing patient-centered training, protocols, and scripts to increase comfort in providing SBI. Through this qualitative study, we have obtained critical stakeholder data that can be used to design SBI in future research.

Patients in our sample had long-term experience with opioids, with issues related to medication access. This is similar to other study findings that show recent opioid prescribing guidelines [25] may have led to inadequate pain management [2]. Patients in our study did not trust healthcare professionals when they discussed opioid tapering. Research suggests that lack of trust in healthcare professionals does not promote optimal pain care [26] and may be exacerbated by prevention interventions that are not patient-centered and focus solely on reducing prescribing rates [27]. These are important considerations for future SBI design.

Despite their long-term experience taking opioids, there was a severe lack of knowledge regarding opioid safety among patients, with ‘take as prescribed’ being the only direction provided to them. Patients reported using informal and unverified sources of information such as the internet or other patients. Research indicates that this lack of opioid safety knowledge, especially related to overdose risks and naloxone, is very common among patients with chronic pain [28, 29]. As most harm reduction efforts are targeted towards people using illicit drugs, patients using prescribed opioids may have lower knowledge regarding opioid safety [30]. These findings indicate that patient education, irrespective of opioid misuse behaviors, is important for future SBI design.

Beliefs such as not being at risk of opioid misuse, overdose, or developing OUD were also very common. Pharmacists believed this led to patients practicing risky behaviors such as storing large quantities of opioids and refusing naloxone. Research suggests that individuals who believe that opioid addiction risk is personally irrelevant have a higher risk of opioid misuse [31]. However, patients in our sample were comfortable with pharmacists providing information about opioid safety as part of SBI, if done in a non-stigmatizing manner.

 Patients described needing education on long-term opioid use and recognizing opioid dependence along with patient-centered opioid safety knowledge. These needs could be met as part of patient-centered counseling (BI), ideally at index prescription when patients may be most receptive. A recent web-based digital intervention that met some of these needs increased patient knowledge and was rated as highly acceptable by patients [32].

Most patients described being stigmatized by healthcare professionals, including pharmacists when accessing opioid medications. Although few pharmacists openly discussed having bias towards patients in our interviews, many mentioned concerns about coming across as stigmatizing. Research indicates that pharmacists commonly distance themselves from patients who misuse opioids and hesitate to form therapeutic relationships with them [33]. While all patients were comfortable with the pharmacist providing opioid related information, very few had the experience of receiving patient-centered counseling regarding opioid safety. Research suggests that stigma is a barrier to participation in opioid-related interventions for both groups because patients are wary of feeling interrogated or labeled, and pharmacists are wary of making patients uncomfortable [34]. Pharmacists may require anti-bias training and patient-centered education. Such trainings have been shown to increase pharmacist knowledge about opioid misuse and decrease stigma [35]. Packaging SBI as a value added clinical service for all patients taking opioids may also help improve the patient-pharmacist interaction. Future studies should evaluate these strategies to design effective SBI.

Both groups were comfortable with a short self-reported screening tool, in addition to routine practice (using PDMP and technician help). This model that has been studied previously [36], where standardized tools such as the Prescription Opioid Misuse Index, [36, 37] the Opioid Risk Tool [38–41], or the Routine Opioid Outcome Monitoring tool [42, 43] were used. These studies also show promising potential for effectiveness of pharmacy-based SBI for opioids. Both groups also expressed support for pharmacy-based SBI focused on patient education regarding both opioid safety as well as general chronic opioid use, regardless of misuse behaviors. Since most opioid safety initiatives are not designed to be universal prevention [30], such SBI could potentially fill the gap in a patient population that is often overlooked. However, in busy large-chain pharmacies or those without private space, alternate formats of counseling such as telephone-based, telehealth, or digital applications may be more feasible and acceptable [44, 45].

Participants also discussed naloxone and contacting prescribers as potential brief interventions. A recent pharmacy-based SBI has found some success in increasing naloxone uptake [38–41]. However, pharmacists may need non-stigmatizing scripts focused on patient autonomy [46]. While pharmacists contacting prescribers could potentially reduce inappropriate prescriptions, research indicates that prescriber-pharmacist relationships and communication are often tense, ineffective, and a barrier to improving pharmacist roles in OUD prevention and treatment [47, 48]. Pharmacists in our study suggested that stakeholder engagement with prescribers to ensure their support of SBI may be needed.

Patients described needing a SBI delivery format that offers privacy and autonomy. Pharmacists needed a protocol and training to be able to efficiently provide SBI. Lack of time, role limitations, and stigma/privacy were the main implementation challenges. Research suggests that these role limitations hamper pharmacists’ self-efficacy in providing opioid safety services [13]. These challenges could potentially be overcome by offering alternative formats such as digital SBI, training pharmacists, fitting intervention within pharmacy workflows, and marketing SBI as a clinical service. Such strategies can be included in designing SBI in future research.

This study has some limitations. Patient interviews were conducted with a sample diverse in terms of pain chronicity and pharmacy experience but most patients were white, had insurance, and lived in suburban areas. As health disparities regarding opioids and OUD treatment are common in racial and ethnic minority groups, underinsured, and more rural populations, involving patients from these groups could lead to different themes. Therefore, findings from the patient interviews cannot be transferred to all patients using opioids. Future research should focus on engaging these groups individually and developing SBI that target their specific needs rather than a one-size-fits-all approach. Our study focused only on the screening and brief intervention portion of the SBIRT model. Referral to treatment is an important component that was not explored thoroughly in our study.

## Conclusion

In this implementation-focused qualitative study comparing patient and pharmacist views on opioid misuse SBI, we found that patients needed education on opioid safety and general opioid use, regardless of misuse behaviors. Pharmacists described the need for patient-centered training, protocols, and scripts to provide SBI. A short-self-reported screening and brief interventions including counseling, naloxone, and involving prescribers were discussed by both groups. Alternate formats of SBI using digital health technologies may be needed for effective design and implementation.

### Supplementary Information

**Additional file 1: **CFIR Constructs.

**Additional file 2: **Sample interview questions.

**Additional file 3: **COREQ Checklist.

## Data Availability

Data sharing is not applicable to this article as no datasets were generated or analyzed during the current study.
