# Supplementary material for: Patient and pharmacist perspectives on opioid misuse screening and brief interventions in community pharmacies
Source: Addict Sci Clin Pract. 2024 Apr 8;19:27. doi: 10.1186/s13722-024-00460-y (PMC11003152; doi:10.1186/s13722-024-00460-y)
Supplement: Supplementary file 1 — Additional file 1: CFIR Constructs. [file 13722_2024_460_MOESM1_ESM.docx]

Table: CFIR Constructs (with bolded constructs included in the study)

| Innovation Attributes | Inner Setting | Outer Setting | Characteristics of Individuals | Processes |
| --- | --- | --- | --- | --- |
| Source | **Structural characteristics** | **Patient needs and resources** | **Knowledge & beliefs** | **Planning** |
| Evidence strength and quality | **Network/ Communication** | Peer pressure | **Self-Efficacy** | **Engaging** |
| **Relative advantage** | **Culture** | Cosmopolitism | Stage of change | Implementing |
| Intervention:  **Adaptability**  Trialability  **Complexity**  Design quality & packaging  **Cost** | Implementation Climate  **Change Tension**  **Compatibility**  **Relative Priority**  **Organizational incentives**  **Goals & feedback**  Learning climate | External policies and incentives | Personal attributes:  Ambiguity tolerance  **Motivation**  Values  Competence  Capacity  Learning style  Innovativeness | Evaluating |
|  | Readiness for implementation |  | Individual identification with organization |  |
