# Supplementary material for: Patient and pharmacist perspectives on opioid misuse screening and brief interventions in community pharmacies
Source: Addict Sci Clin Pract. 2024 Apr 8;19:27. doi: 10.1186/s13722-024-00460-y (PMC11003152; doi:10.1186/s13722-024-00460-y)
Supplement: Supplementary file 2 — Additional file 2: Sample interview questions. [file 13722_2024_460_MOESM2_ESM.docx]

Table: Interview Questions for Pharmacists and Patients

| **CFIR Domain** | **Constructs** | **Pharmacist Questions^#^** | **Patient Questions^#^** |
| --- | --- | --- | --- |
| Innovation Attributes | Relative advantage | How does the SBI compare to other similar existing programs in your pharmacy? | What other pharmacy-based programs for opioid medicines do you know about? How does this compare? |
|  | Adaptability | What kinds of changes do you think you will need to make to the SBI so it will work effectively in your pharmacy? | What kinds of changes would you prefer in the program so it will work effectively for you? |
|  | Complexity | How complicated is it to provide the SBI? How can it be made simpler? | What barriers do you think will stop patients from participating in the program? How complex is this program? |
|  | Cost | What costs will be incurred to implement the SBI? How do costs compare to benefits? | What are some possible ways this program may be beneficial? What disadvantages do you see in participation? |
| Inner Setting | Structural characteristics | What kinds of infrastructure changes will be needed to accommodate the intervention? | How should the program be conducted in your pharmacy, for you to comfortably participate in the program? |
|  | Network/ Communication | Can you describe your working relationships with your colleagues? With managers/leaders?  How do you typically find out about new information?  When you need to solve a problem, what do you do? Who are your "go-to" people? | When you have questions about your opioid medicines, what steps do you take to seek out answers?  Have you ever talked with your pharmacist about opioid medications?  If yes, what has your experience been in communicating with your pharmacist about opioid medicines?  If not, what inhibits your willingness to talk with your pharmacist? |
|  | Culture | How would you describe the culture of your setting? To what extent are new ideas embraced and used to make improvements in your organization? | N/A |
|  | Change Tension | Is there a strong need for this intervention? How essential is this intervention to meet the needs of the patients? | N/A |
|  | Compatibility | How well does the intervention fit with existing work processes and practices in your setting? | How do you feel about this program being conducted in your local community pharmacy? |
|  | Organizational incentives | What incentives would you need to provide the intervention? | N/A |
|  | Goals & feedback | How does the SBI align with your organization goals? | N/A |
| Outer Setting | Patient needs and resources | How well does the SBI meet the needs of patients? | What do you need to help you take your opioid medicine safely? How well would this program meet your needs? |
| Characteristics of Individuals | Knowledge & beliefs | What do you know about SBIs? How do you feel about SBI for opioid misuse implemented in your pharmacy? | What do you know about taking opioid medicines safely? How do you feel about pharmacists talking to you about opioid medicines? |
|  | Self-Efficacy | How confident are you that you will be able to provide the SBI? | How confident are you in taking your opioid medicines correctly? How can pharmacists improve your confidence? |
|  | Motivation | What would motivate you to provide the SBI? | If such a program is developed, would you be interested in participating? Why? |

# Order of the interview questions was changed to maintain flow in the final interview guide
